# Advancing Behavioral Medicine: The International Pursuit of Science for 30 Years

**DOI:** 10.1007/s12529-024-10330-1

**Published:** 2024-10-31

**Authors:** Michael A. Hoyt, Ren Liu, Chun-Qing Zhang

**Affiliations:** 1 University of California, Irvine, 856 Health Sciences Drive, Irvine, CA 92697-3957 USA; 2 University of California, Merced, Merced USA; 3 Sun Yat-Sen University, Guangzhou, China

**Keywords:** Behavioral medicine, Systematic review, Meta-analysis

This special issue commemorates the 30-year anniversary of the *International Journal of Behavioral Medicine* (*IJBM*), the official scientific journal of the International Society for Behavioral Medicine (ISBM). In celebration of this momentous milestone, this issue of *IJBM* presents systematic, scoping, and integrative literature reviews and meta-analyses that synthesize the state of the science in foundational areas of the field. Answering this call, contributing authors brought strong focus on intervention research across broad areas of behavioral medicine. We are heartened by the focus on evaluating, refining, and optimizing interventions based on a careful and wide-ranging review of the scientific knowledge base.

The included reviews and meta-analyses effectively aggregate diverse findings and offer a comprehensive understanding of interventions across various populations and settings. Behavioral medicine has made strong empirical, theoretical, and clinical contributions to the understanding and treatment of chronic pain conditions. Both Wirtz et al. [1] and Gnall et al. [2] offer useful reviews of chronic pain interventions that highlight innovative and forward-thinking considerations. Gnall et al. [2] evaluate the use of mind–body therapies that include intervention approaches such as movement modalities (e.g., tai chi), mindfulness, and self-compassion meditation to impact interoception. Behavioral medicine has always been on the forefront of discovery of non-pharmacologic approaches and the use of scientific evidence to guide clinical care. Their focus on a mechanistic outcome is a wise use of meta-analysis to guide the research toward more optimized use of this collection of emerging interventions for pain control. Wirtz et al. [1] focus on a specific patient population to evaluate the evidence of a small but important literature. Their finding that integrative interventions have stronger evidence for effectiveness than purely biomedical treatments calls forth the place for behavioral medicine in the landscape of patient care and the treatment of chronic conditions.

Behavioral medicine’s contributions to supportive cancer care are noteworthy. Two reviews in this issue reflect on the field’s accomplishments in behavioral oncology and point us to important future directions. Llave et al. [3] use meta-analyses to evaluate the state of the field on interventions for individuals with advanced cancer or metastatic disease. This important review simultaneously highlights the impact of behavioral medicine interventions in the improvement of cancer survivorship outcomes while shedding light on the need for strong attention on those patients with advanced illness. Moreover, they provide an exemplar for future reviews to code for cultural considerations particularly as they apply to intervention design and assessment and measurement. The review by Marzilliano and colleagues [4] also emphasizes the importance of measurement in behavioral medicine research with a focus on the evaluation of measures of social isolation and loneliness in cancer research. Heightened by our global experiences of the Covid-19 pandemic, the importance of social isolation to our health, particularly for those with complex illnesses and treatment plans, should remain a paramount priority in the years ahead.

The meta-analyses by Mass genannt Bermpohl et al. [5] focused on the acceptance of cognitive therapy interventions by individuals with chronic fatigue syndrome and that by Wei et al. [6] on quality-of-life interventions for patients with psoriasis. Both of these studies guide us forward in developing more effective interventions as well as provide strong examples of how meta-analyses can help inform literature across the translational spectrum. Mass genannt Bermpohl and colleagues [5] focus on outcomes particularly valuable in earlier phase research (e.g., intervention completion rates, participant drop-out, impact of time in therapy). More emphases on factor relevant to phase I and II research will better inform future intervention design. Wei and colleagues [6] provide us an example of how meta-analysis can reveal unexpected findings. They find support for behavioral medicine interventions with patients with psoriasis on decreasing anxiety, but not for depression and overall quality-of-life.

This issue also includes examples of how systematic reviews can be used to synthesize knowledge to optimize utility. Klink et al. [7] offer a qualitative synthesis of dietary recommendation messaging. Qualitative research methods are widely used across the field of behavioral medicine and enrich the knowledge base with in-depth, thematic, and often theoretically rich findings. Synthesis of qualitative studies reveals shared elements across the literature and identification of broad themes, and as demonstrated in their study, can result in identification of implication-rich and meaning-laden conclusions. Similarly, Sola et al. [8] give us a review of reviews regarding cognitive performance in individuals with type 2 diabetes. As demonstrated, this type of synthesis uses prior published reviews as the unit of analysis. As the field of behavioral medicine continues to mature, review of reviews will become increasingly valuable in tracking the current state of knowledge and practice.

Both Zhang et al. [9] and Kompf and Rhodes [10] focus on physical activity. Again, these reviews provide support and opportunity for effective use of behavioral interventions. Zhang et al. [9] reviewed and evaluated the use of digital behavior change interventions aimed at reducing sedentary behavior in adults with diabetes. Their analyses provide evidence for the potential of digitally delivered interventions, and of particular importance, reveal effects of specific behavior change techniques that impact behavior. Kompf and Rhodes [10] conducted a systematic review on resistance training behavior with a focus on evaluation of mediating processes (e.g., capability, motivation, opportunity). These reviews point to the valuable place of behavior change theory in behavior medicine intervention. For instance, social cognition models have been widely used to understand the psychosocial determinants of health behaviors [11]. Notably, Zhang and colleagues [9] reported that most of the digital behavioral interventions on reducing sedentary behavior and promoting physical activity in adults with diabetes were theory-driven, with Social Cognitive Theory [12, 13] being the most commonly reported. Likewise, Kompf and Rhodes [10] also found Social Cognitive Theory [12, 13] to be the most used theory in their review of interventions to change resistance training followed by Self-Determination Theory [14, 15].

Theory-driven behavioral medicine interventions can lead to meaningful change in health behavior [16–18]. However, we note that only two out of 11 reviews in this issue substantively explored the role of theory in the studies they reviewed. Interventions that lack a theoretical ground may impede progress on identifying the content of these interventions that are effective in changing preventive behaviors and even blur our ability to understand why and when intervention efficacy does not generalize across contexts, behaviors, and populations [19]. These papers highlight that identifying the theory-based components of behavioral interventions, and testing their efficacy, is most likely to provide evidence for which techniques or mechanisms are most likely to be effective [20].

The papers in this issue demonstrate how aggregated data can yield actionable insights for behavioral medicine. By analyzing multiple randomized controlled trials, researchers can assess the overall efficacy of interventions and identify specific components that contribute to success. At the same time, one should point to the many challenges that authors face that are inherent in conducting reviews and meta-analyses in behavioral medicine. A prominent concern is the variability in study quality and design. Included studies across these meta-analyses and reviews utilize different measurement tools, vary widely in sample sizes, and include great variation in intervention protocols, leading to heterogeneity that complicates the synthesis of results. For example, while some studies on physical activity interventions may rely on self-reported data, others might utilize more objective measures. The introduction of such variability can skew overall effect sizes. Furthermore, publication bias remains a persistent concern. Studies with statistically significant results are more likely to be published, creating a skewed representation of the efficacy of interventions. Addressing this bias requires efforts to incorporate unpublished data and promote transparency in research reporting. An additional challenge is the need for context-sensitive interpretations of findings. While meta-analyses can provide a broad overview of trends, they may overlook the cultural, socioeconomic, and environmental factors that influence the effectiveness of interventions in specific populations. For instance, the implementation of dietary interventions in high-income countries may not be directly applicable to low-income settings without consideration of local food systems, health beliefs, and access to resources.

Looking forward, the role of systematic reviews and meta-analyses in behavioral medicine is poised to evolve significantly. Advances in technology and data analytics are likely to enhance the precision of these methodologies. The integration of big data and machine learning could enable researchers to conduct more nuanced subgroup analyses, identifying specific factors that moderate intervention effectiveness across diverse populations. Additionally, incorporating qualitative methodologies into systematic reviews can enrich the narrative of behavioral interventions, offering insights into the lived experiences of individuals undergoing treatment. This mixed-methods approach can foster a deeper understanding of the barriers and facilitators to behavior change, thus informing more effective, culturally sensitive interventions. Through this special issue, we aim to pave the way for future meta-analyses in the dynamic field of behavioral medicine.

The strong focus on intervention research in this special issue underscores the impact the field of behavioral medicine can have on the improvement of health and reduction of risk. It can also reveal the opportunities for innovative new directions. Notably, the preponderance of interventions reviewed across these reviews is at the individual level. Few, if any, target organizational-, community-, or policy-level changes. Multi-level interventions provide a possible future direction for future work. In this issue, Persad-Clem and colleagues [21] give us a review of the use of community engagement in behavioral medicine. This includes the engagement of community partners in behavioral medicine research, the use of community-based participatory research methods, and engagement of key stakeholders. In addition to reviewing the state of the literature, this article serves as a primer by providing a strong set of recommendations for the field in the engagement of community partners and the strengthening of intervention development, testing, and implementation.

In summary, over the past 30 years, *IJBM* has found its place in the broad, growing, and innovative field of behavioral medicine. The journal has offered a scientific forum for the international community of researchers and practitioners. As evidenced by this special issue, the state of the field is strong. The next 30 years holds significant promise.
